# Color, Music, and Emotion: Bach to the Blues

**DOI:** 10.1177/2041669518808535

**Published:** 2018-11-11

**Authors:** Kelly L. Whiteford, Karen B. Schloss, Nathaniel E. Helwig, Stephen E. Palmer

**Affiliations:** Department of Psychology, University of Minnesota, Minneapolis, MN, USA; Department of Psychology, University of Wisconsin–Madison, Madison, WI, USA; Wisconsin Institute for Discovery, University of Wisconsin–Madison, Madison, WI, USA; Department of Psychology, University of Minnesota, Minneapolis, MN, USA; School of Statistics, University of Minnesota, Minneapolis, MN, USA; Department of Psychology, University of California, Berkeley, CA, USA

**Keywords:** aesthetics, color, music cognition, emotion, cross-modal associations

## Abstract

When people make cross-modal matches from classical music to colors, they choose colors whose emotional associations fit the emotional associations of the music, supporting the *emotional mediation hypothesis*. We further explored this result with a large, diverse sample of 34 musical excerpts from different genres, including Blues, Salsa, Heavy metal, and many others, a broad sample of 10 emotion-related rating scales, and a large range of 15 rated music–perceptual features. We found systematic music-to-color associations between perceptual features of the music and perceptual dimensions of the colors chosen as going best/worst with the music (e.g., *loud, punchy, distorted* music was generally associated with *darker, redder, more saturated* colors). However, these associations were also consistent with emotional mediation (e.g., *agitated*-sounding music was associated with *agitated*-looking colors). Indeed, partialling out the variance due to emotional content eliminated all significant cross-modal correlations between lower level perceptual features. Parallel factor analysis (Parafac, a type of factor analysis that encompasses individual differences) revealed two latent affective factors—***arousal*** and ***valence***—which mediated lower level correspondences in music-to-color associations. Participants thus appear to match music to colors primarily in terms of common, mediating emotional associations.

## Introduction

Music–color synesthesia is a rare and interesting neurological phenomenon in which listening to music automatically and involuntarily leads to the conscious experience of color (Ward, 2013). Although only a small proportion of people have such synesthesia, recent evidence suggests that self-reported nonsynesthetes exhibit robust and systematic music-to-color associations (e.g., Isbilen & Krumhansl, 2016; Lindborg & Friberg, 2015; Palmer, Schloss, Xu, & Prado-Leon, 2013; Palmer, Langlois, & Schloss, 2016). For example, when asked to choose a color that “goes best” with a classical musical selection, both U.S. and Mexican participants chose lighter, more saturated, yellower colors as going better with faster music in the major mode (Palmer et al., 2013).

Two general hypotheses have been proposed to explain such music-to-color associations in both synesthetes and nonsynesthetes: direct links and emotional mediation. The *direct link hypothesis* asserts that musical sounds and visual colors are related via direct correspondences between the perceived properties of the two types of stimuli (e.g., Caivano, 1994; Pridmore, 1992; Wells, 1980). For example, Caivano (1994) proposed that the octave-based musical scale maps to the hue circle, luminosity to loudness, saturation to timbre, and size to duration. Pridmore (1992) suggested that hue maps onto tone via wavelength and that loudness maps to brightness via amplitude. Lipscomb and Kim (2004) found that pitch mapped to vertical location, timbre with shape, and loudness with size. A well-documented example is that higher pitched tones are associated with lighter, brighter colors (e.g., Collier & Hubbard, 2004; Marks, 1987; Ward, Huckstep, & Tsakanikos, 2006).

In contrast, the *emotional mediation hypothesis* suggests that color and music are related indirectly through common, higher level, emotional associations (Barbiere, Vidal, & Zellner, 2007; Bresin, 2005; Lindborg & Friberg, 2015; Palmer et al., 2013, 2016; Sebba, 1991). In this view, colors are associated with music based on shared emotional content. For example, happy-sounding music would be associated with happy-looking colors and sad-sounding music with sad-looking colors. Most evidence for emotional mediation comes from studies in which nonsynesthetes were asked to choose colors according to how well they went with different selections of classical music (Isbilen & Krumhansl, 2016; Palmer et al., 2013, 2016). Participants subsequently rated each musical selection and each color on several emotion-related scales^1^ (e.g., *happy/sad, angry/calm, strong/weak*), so the role of emotion in their color–music associations could be assessed. Results showed strong correlations between the emotion-related ratings of the musical excerpts and the emotion-related ratings of the colors people chose as going best/worst with the music (e.g., +.97 for *happy/sad* and +.96 for *strong/weak*; Palmer et al., 2013).^2^ Similarly, strong correlations were found for classical music even when using highly controlled, single-line piano melodies by Mozart (e.g., +.92 for *happy/sad* and +.85 for *strong/weak*; Palmer et al., 2016). These results were corroborated for Bach preludes (Isbilen & Krumhansal, 2016) and film music (Lindborg & Friberg, 2015) using different methods and analytic techniques.

Note that emotional mediation does not imply that the music–perceptual features are irrelevant to people's color associations. Indeed, combinations of the music's perceptual features (e.g., subjective qualities such as *loudness* and *rhythmic complexity*) carry information that helps determine the emotional character of the music (Friberg, Schoonderwaldt, Hedblad, Fabiani, & Elowsson, 2014), but this emotional interpretation may be influenced by experience, such as cultural differences (e.g., Gregory & Varney, 1996). For example, major/minor mode is important in conveying *happy/sad* emotions in music for Western listeners (e.g., Parncutt, 2014), but this does not necessarily mean that music in the major mode maps directly to colors that are bright and saturated in the absence of *happy* emotions.

This study was designed to further investigate the generality of the emotional mediation hypothesis by extending previous studies in four main ways. First, we expanded the diversity of musical excerpts by studying music within 34 (author-identified) genres, including Salsa, Heavy metal, Hip-hop, Jazz, Country-western, and Arabic music, among others (Table 1). Second, to better describe this wider variety of music, we expanded the music–perceptual features to include a more diverse set of 15 rating scales, including *loudness, complexity, distortion, harmoniousness*, and more (Table 2(a)). We then examined how color–appearance dimensions of the colors (bipolar scales of *saturated/desaturated, light/dark, red/green*, and *yellow/blue*) picked to go with the music relate to these music–perceptual features. Third, we expanded the set of emotion-related scales to a wider range including *appealing/disgusting, spicy/bland, serious/whimsical*, and *like/dislike* (Table 2(b)). We included preference (*like/dislike*) because preferences play a significant role in cross-modal odor-to-color pairings (Schifferstein & Tanudjaja, 2004)—people tend to associate colors they *like/dislike* with odors they *like/dislike*—and we are not aware of any research showing if that effect generalizes to music-to-color associations. However, evidence suggests that congruent music-to-color associations increase aesthetic preferences (Ward, Moore, Thompson-Lake, Salih, & Beck, 2008). Finally, the present data analyses employ more powerful analytic techniques, including partial correlations to test the emotional mediation hypothesis and the parallel factor model (Parafac; Harshman, 1970) to understand the underlying affective factors involved.

**Table 1.** The 34 Musical Excerpts.

Genre	Sat.	L/D	Y/B	R/G
1(Alternative)	139	39	9	20
2(Arabic)	104	−24	17	44
3(Bach)	−4	62	0	−19
4(Balkan Folk)	−29	26	−5	−22
5(Big Band)	−50	19	−4	−18
6(Bluegrass)	37	72	79	−40
7(Blues)	34	−46	−44	−33
8(Classic Rock)	65	−91	13	38
**9(Country Western)**	−27	92	65	−48
10(Dixieland)	98	25	19	36
**11(Dubstep)**	57	−116	−35	29
12(Eighties Pop)	103	90	22	0
13(Electronic)	70	−42	−25	1
14(Folk)	−27	96	−6	−54
15(Funk)	66	−62	−31	4
16(Gamelon)	21	25	21	−25
17(Heavy Metal)	−21	−190	−31	44
18(Hindustani Sitar)	69	−44	15	26
19(Hip Hop)	26	0	−55	0
**20(Indie)**	−56	85	−29	−52
**21(Irish)**	88	40	18	−63
22(Jazz)	86	9	−33	5
**23(Mozart)**	−6	124	−7	−24
**24(Piano)**	−129	0	−103	−60
25(Progressive House)	141	−36	−9	50
26(Progressive Rock)	31	78	−35	−27
27(Psychobilly)	79	−126	−25	39
28(Reggae)	58	41	−13	−37
**29(Salsa)**	212	34	60	76
**30(Ska)**	180	42	30	32
31(Smooth Jazz)	14	84	−27	−6
32(Soundtrack)	−5	88	−39	−51
33(Stravinsky)	41	3	9	16
34(Trance)	148	−17	−10	51

*Note*. All numbers correspond to the weighted average color–appearance ratings of the colors picked to go with the music (PMCA scores), averaged across subjects (details described in the subsection “Results of lower-level perceptual correlations in music-to-color associations” of the “Results and Discussion” section and Appendix A). Entries in boldface indicate selections for which first-choice colors are presented in Figure 2. Sat. = saturation; L/D = light(+)/dark(−); Y/B = yellow(+)/blue(−); R/G = red(+)/green(−).

**Table 2.** The Music–Perceptual Features (a) and Emotion-Related Scales (b).

(a) Music–perceptual features	
1. Electric/Acoustic	
2. Distorted/Clear	
3. Many/Few instruments	
4. Loud/Soft	
5. Heavy/Light	
6. High/Low pitch	
7. Wide/Narrow pitch variation	
8. Punchy/Smooth	
9. Harmonious/Disharmonious	
10. Clear/No melody	
11. Repetitive/Nonrepetitive	
12. Complex/Simple rhythm	
13. Fast/Slow tempo	
14. Dense/Sparse	
15. Strong/Weak beat	
(b) Emotion-related scales	
1. Happy/Sad	
2. Calm/Agitated	
3. Complex/Simple	
4. Appealing/Disgusting	
5. Loud/Quiet	
6. Spicy/Bland	
7. Warm/Cool	
8. Whimsical/Serious	
9. Harmonious/Dissonant	
10. Like/Dislike	

*Note*. See Supplementary Data1-4.csv for across-subject averages.

## Methods

### Participants

Three independent groups of participants all gave written informed consent and received either course credit or monetary compensation for their time. The experimental protocol was approved by the Committee for the Protection of Human Subjects at the University of California, Berkeley (approval #2010-07-1813).

#### Group A

Thirty participants (18 females) performed Tasks A1 to A4 described later. All had normal color vision, as screened with the Dvorine Pseudo-Isochromatic Plates, and none had any form of synesthesia, as assessed by the initial Synesthesia Battery questionnaire (Eagleman, Kagan, Nelson, Sagaram, & Sarma, 2007).

#### Group B

Fifteen musicians (four females), who were members of either the University of California Marching Band or the University of California Wind Ensemble, provided ratings for the musical excerpts in terms of 15 global musical features, as described in Task B1.

#### Group C

The color–perceptual ratings data from 48 participants from Palmer et al. (2013) were used in evaluating the present results (see Task C1).

### Design and Stimuli

#### Colors

**Figure 1.**
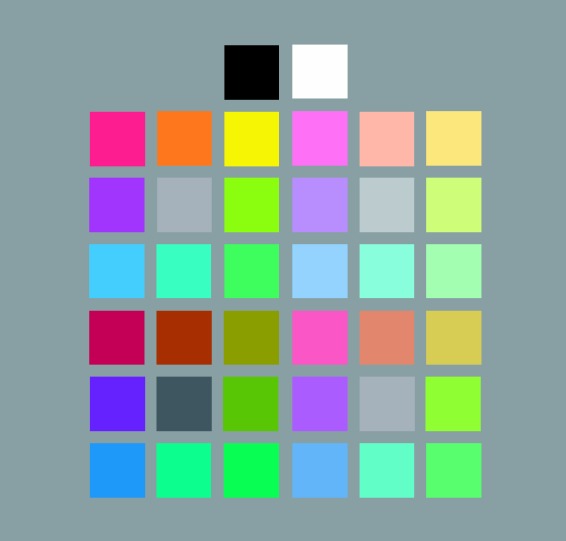
The 37 colors used in the experiment (from Palmer et al., 2013). The top left and bottom right gray appeared twice for consistency with the stimulus design and with Palmer et al. (2013).

The colors were the Berkeley Color Project 37 (BCP-37) colors studied by Palmer et al. (2013) (Figure 1; Table S1 in Supplementary Materials for CIE 1931 xyY and Munsell coordinates). The colors included eight hues (red (R), orange (O), yellow (Y), chartreuse (H), green (G), cyan (C), blue (B), and purple (P)) sampled at four “cuts” (saturation/lightness levels): saturated (S), light (L), muted (M), and dark (D). The colors were initially sampled from Munsell space (Munsell, 1966), with the goal of obtaining highly saturated colors (S) within each hue, and then sampling less-saturated versions of those hues at varying lightness levels—light (L), medium (M), and dark (D). The Munsell coordinates were translated to CIE 1931 xyY coordinates using the Munsell Renotation Table (Wyszecki & Stiles, 1982). The S-colors for each hue were the colors with the highest saturation that could be displayed on the computer monitor used by Palmer et al. (2013) for each hue. The lightness (value) of the S-colors differed across hues because the most saturated versions of each hue occur at different lightness levels across hues, due to the nature of the visual system (Wyszecki & Stiles, 1982). In Munsell space, the L-colors were approximately halfway between S-colors and white, M-colors were approximately halfway between S-colors and neutral gray, and D-colors were approximately halfway between S-colors and black. Therefore, the L-, M-, and D-colors had the same Munsell chroma within each hue, and their chroma and value were scaled relative to the S-color of each hue.

There were also five achromatic colors: white (WH), black (BK), light gray (A_L_), medium gray (A_M_), and dark gray (A_D_). In this notation, “A” stands for achromatic, and the subscript stands for light (L), medium (M), and dark (D). A_L_ had approximately the average luminance of all eight L-colors, A_M_ had approximately the average luminance of all eight M-colors (and of the S-colors), and A_D_ had approximately the average luminance of all eight D-colors. The colors were always presented on a gray background (CIE x = 0.312, y = 0.318, Y = 19.26).

In the music-to-color association task, the 37 colors were displayed on the screen in the spatial array shown in Figure 1, with each color displayed as a 60 × 60 pixel square.^3^ All visual displays were presented on a 21.5 in. iMac computer monitor with a resolution of 1680 × 1050 pixels using Presentation software (*www.neurobs.com*). In tasks that displayed the 37 colored squares, the task was completed in a dark room. The monitor was characterized using a Minolta CS100 Chromometer to ensure that the correct colors were presented. The deviance between the target color's CIE xyY coordinates (Table S1) and its measured CIE xyY coordinates was < .01 for x and y and less than 5 cd/m^2^ for Y.

#### Music

The 34 musical stimuli were instrumental excerpts from 34 different genres (Table 1 and Table S2). The primary goal of the selection procedure was to use a more diverse sample of music than in previous studies (Isbilen & Krumhansl, 2016; Palmer et al., 2013, 2016). The first, second, and last authors chose excerpts that (a) contained no lyrics (to avoid contamination by the meaning of the words), (b) were unlikely to be familiar to our undergraduate participants, (c) conveyed a range of different emotions, and (d) were musically distinct, so that no two selections sounded too similar.^4^ None of these selections should be interpreted as standing for the entire genre used to label them, but only as single examples that were chosen to achieve a diversity of excerpts that come from different (author-identified) genres. Because musical genres are highly variable, they span wide ranges of variation that cannot be represented by any single example.

The same authors chose the names used in referring to the genres and the excerpts, which were never displayed or mentioned to the participants. In most instances, the genre name corresponded to the genre the artist affiliated with their music on their website or album or the genre label of the given musical selection on iTunes. The exceptions were two musical excerpts that were labeled by the dominant timbre (Gamelan and Piano), as well as the three classical pieces, which were labeled with the name of the composer—Mozart, Bach, and Stravinsky—to differentiate easily among them. We make no claim that the genre-excerpts studied were sampled systematically from among all forms of music, only that the present musical excerpts were more diverse than stimuli used in several previous studies examining music-to-color associations.

The musical excerpts were edited using Audacity software (*audacity.sourceforge.net)* by clipping a 15-s excerpt and adding a 2-s fade-in and fade-out. All musical excerpts were presented through closed-ear headphones (Sennheiser Model HD 270). The level was determined by having a different set of 19 participants listen to the 34 excerpts through the same headphones and adjust the volume “to the appropriate loudness level” for that musical selection. The level of each musical selection for the main experiment was determined by the average data from this task. This method was used to present the excerpts at more natural, ecologically valid listening levels than if the level had been constant across musical stimuli.

The emotion-related scales were selected from a larger set of 40 scales, with the purpose of retaining 10 scales that were distinct from one another and relevant for both colors and music, based on pilot data from *n* = 28 participants (see S1 Text). The 15 music–perceptual features were selected by examining the music cognition literature (e.g., Rentfrow et al., 2012) and choosing any feature that seemed potentially relevant to the 34 musical excerpts in the experimenters' collective judgment (Table 2(b)).

### Experimental Tasks

#### Overview

Six tasks were performed by the three groups of participants as described below and summarized in Table 3.

**Table 3.** Summary of the Six Tasks.

Task	Group	Task summary
A1: Music-to-color associations	A: 30 participants	Participants chose the three best and three worst colors among 37 colors (Figure 1) to go with each of the 34 musical excerpts (Table 1)
A2: Color–emotion ratings		Participants rated each of the 37 colors on each of the 10 emotion-related scales (Table 2(b))
A3: Music–emotion ratings		Participants rated each of the 34 musical excerpts on each of the 10 emotion-related scales (Table 2(b))
A4: Synesthesia questionnaire		Participants completed a subset of questions from the synesthesia questionnaire (www.synesthete.org)
B1: Music–perceptual ratings	B: 15 musicians	Musicians rated each of the 34 musical excerpts on each of the 15 music–perceptual features (Table 2(a))
C1: Color–perceptual ratings	C: 48 participants in Palmer et al. (2013)	Participants rated each of the 37 colors on each of the four color–appearance dimensions (*saturation, light/dark, yellow/blue*, and *red/green*)

##### Task A1: Music-to-color associations

Participants heard the 34 musical excerpts in an individualized random order while viewing the 37-color array. After hearing each full, 15-s selection at least once, participants were asked to choose the three colors that were most consistent with each selection as it was playing. The cursor used to select the colors appeared on the screen after the 15-s excerpt played once, so that participants were required to listen to the entire selection before they could begin selecting colors. They chose the most, second-most, and third-most consistent colors in that order, with each color disappearing as it was selected. After all 37 colors reappeared on the screen, participants were asked to choose the three colors that were most inconsistent with the music, choosing the most, second-most, and third-most inconsistent colors in that order. The music looped continuously until all six color choices had been made for that selection, so that participants could listen to the music as many times as they wished during a given trial. The next musical selection and the full color array were then presented after a delay of 500 ms.

##### Task A2: Color–emotion ratings

Participants then rated each of the 37 colors on each of 10 bipolar emotion-related scales, including their personal preference (Table 2(a)). The preference ratings (*like/dislike*) of all 37 colors were always rated first to avoid being contaminated by the other emotion-related ratings.

Color preferences were rated on a continuous line-mark scale from *Not At All* to *Very Much*. Each color was centered on the screen above the response scale. Participants slid the cursor along the response scale and clicked at the appropriate position to record their response. To anchor the scale prior to making their ratings, participants were shown the entire 37-color array, asked to point to the color they liked the most, and were told that they should rate that color at or near the *Very Much* end point of the scale. They were then asked to point to the color they liked the least and were told that they should rate that color at or near the *Not At All* end point of the scale.

Before performing the other nine emotion-related rating tasks, participants were anchored on each scale by analogous anchoring procedures with appropriate labels at the ends of the bipolar response scale. This anchoring procedure was completed verbally with the experimenter for all nine scales before the subject rated any color on any scale. All nine ratings for one color were made before the next color was presented, and the order of the scales was randomized for each color within participants. The order in which the colors were presented was randomized across participants.

##### Task A3: Music–emotion ratings

Participants rated the same 34 musical excerpts on all 10 emotion-related scales (Table 2(b)) in a manner analogous to that for the colors, with all 34 preference ratings being made before any of the other emotion-related ratings to avoid contamination. The primary difference was that for the anchoring procedures, participants were instructed to recall which previously heard excerpt they thought was, for example, the *happiest* (or the *saddest*), and to click at or near the *happy* (or *sad*) end of the response scale for that selection. The musical excerpts were played one at a time in a random order, and each had to be heard all the way through once before being rated on the scales. All nine scales were rated for one musical excerpt before the next excerpt was presented.

##### Task A4: Synesthesia questionnaire

In the final task, each participant took the Synesthesia Battery questionnaire (Eagleman et al., 2007). If they answered “yes” to any of the questions, they were asked to describe their synesthesia and estimate how frequently it occurs. No data from the experimental tasks were included in the analysis for any participant who answered “yes” to any question.

##### Task B1: Music–perceptual ratings

The 15 musicians rated each musical excerpt on their perception of each of the 15 bipolar, music–perceptual features listed in Table 2(a). Ratings were made in a manner analogous to the emotion-related rating task for music (Task A3). Because these participants had not previously heard the musical excerpts, they all listened to the same representative sample of five musical excerpts before beginning the experiment to exemplify extremes of salient musical features (e.g., the Heavy metal selection was included as an extreme example of *electric, distorted, loud, heavy, low pitch*, and *punchy*, whereas the Piano selection was included as an extreme example of *clear, few instruments, soft, light, high pitch*, and *smooth*). No mention was made of these or any other musical features either before or during the initial presentation of these five selections, however. After listening to all five selections, participants completed an anchoring procedure analogous to the anchoring procedure for the emotion-related rating task for music as described earlier. The 34 excerpts were then presented in a random order and looped continuously until participants completed the task for that excerpt.

##### Task C1: Color–perceptual ratings

The 48 participants described in Palmer et al. (2013) rated the appearance of each of the 37 colors on four color–appearance dimensions—*saturated/desaturated, light/dark, red/green*, and *yellow/blue*—using a 400 pixel line-mark rating scale analogous to those described earlier. The anchoring procedure for each dimension was also analogous to that described earlier for color–emotion ratings (Task A2). Trials were blocked by colors, and the order of the dimensions was randomized within color blocks. The order of colors was randomized across participants.

### Statistical Analysis

All participants completed their given tasks, so there were no missing data points. Across-subject agreement for each rating scale was measured using Cronbach's alpha, and all indicated good-to-excellent consistency (Table S4). Examination of the Q-Q plots of all of the average rating scales suggested the normality assumption did not adhere for some of the scales. To be conservative, all correlations correspond to Spearman's Rho. Parafac does not make any assumptions about the distribution of the data. The only assumption is that the data display systematic variation across three or more modes, which fits well with our three-mode ratings data sets, consisting of stimuli, ratings scales, and subjects.

## Results and Discussion

**Figure 2.**
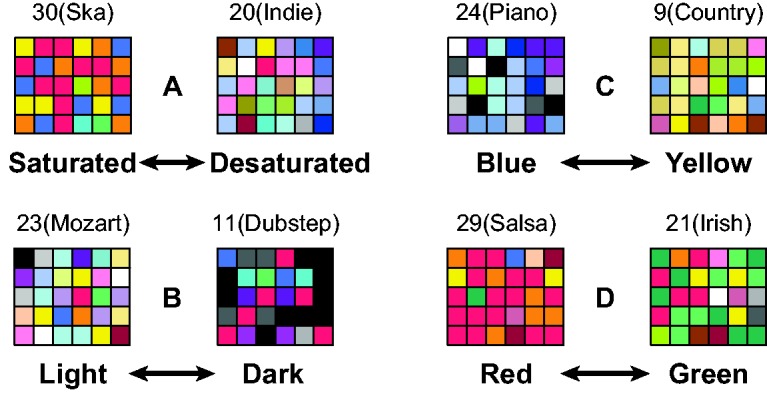
Example of the “best fitting” colors from the cross-modal music-to-color association task. The examples shown represent relatively extreme values for each of the four color–appearance dimensions (*red/green, yellow/blue, light/dark*, and *saturated/desaturated*) with relatively similar values for the other three dimensions. Labels above the array identify the genre name of the musical excerpts represented.

Figure 2 shows the first-choice colors for each of the 30 participants for eight of the musical excerpts. The musical excerpts in Figure 2 were ones that showed particularly strong contrasts along each of the four color–appearance dimensions (see Figure S1 for all 34 musical excerpts). These examples illustrate how participants chose different kinds of colors as going best with different musical excerpts. The colors chosen as going best with the Ska selection, for example, are noticeably more saturated (vivid) than those chosen as going best with the Indie selection, despite the very wide variations in hue for both excerpts. Next, we quantify these differences and address why they might have arisen.

### Results of Lower Level Perceptual Correlations in Music-to-Color Associations

First, we computed 15 across-subject average music–perceptual feature scores (e.g., *loud/soft, fast/slow*) for each excerpt using the ratings from Task B1. These averages were combined with the data from the music-to-color associations (Task A1) and the color–perceptual ratings (Task C1) to compute four Perceptual Music–Color Association scores (henceforth, Perceptual-MCAs or PMCAs) for each of the 34 musical excerpts (for details, see Appendix A). The four Perceptual-MCA scores (four rightmost columns of Table 1) represent the weighted averages of how, *saturated/desaturated, light/dark, red/green*, and *yellow/blue*, the six colors are that were chosen (as the three best and three worst) by each participant.

**Figure 3.**
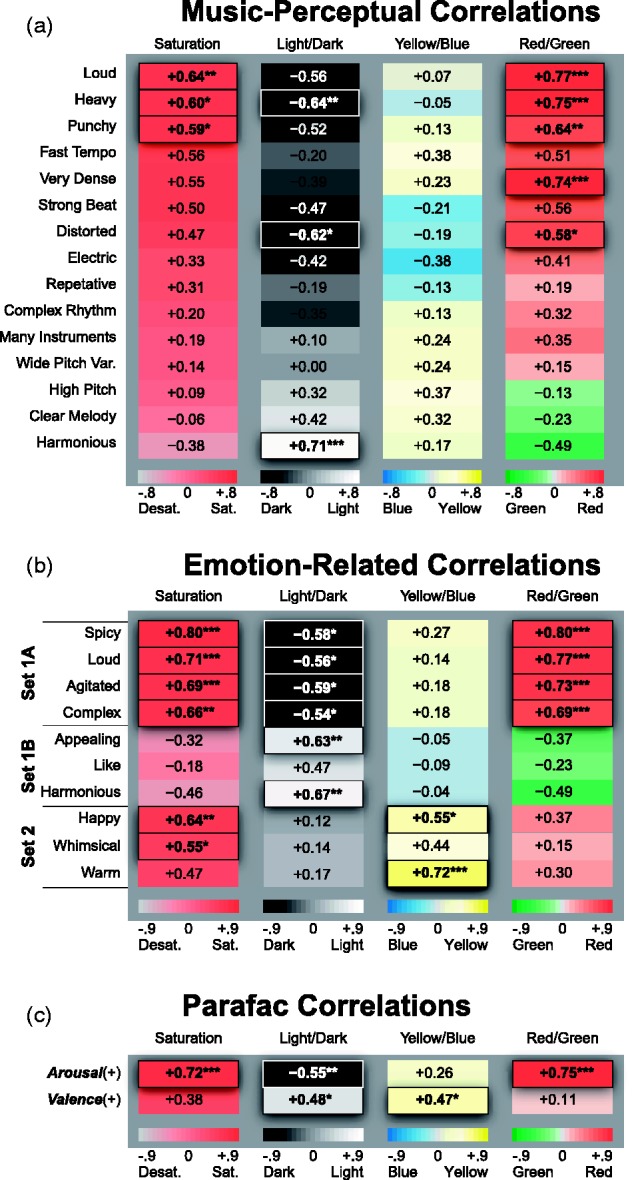
(a) Correlations between the 15 music–perceptual features and the weighted average color–appearance values of the colors picked as going best/worst with the music (the Perceptual-MCAs). (b) Correlations between the 10 music–emotion ratings and the weighted average color–appearance values of the colors picked to go with the music. The grouping of emotion-related features into Sets 1A, 1B, and 2 is explained in the subsection “Results of Higher Level Emotional Correlations in Music-to-Color Associations.” (c) Same correlations as in (b) for the two latent emotion factors identified by Parafac (***arousal*** and ***valence***). Family-wise error rate was controlled using Holm's method. Significant correlations are denoted with asterisks (***p < .001, **p < .01, and *p < .05) and by raised outlined borders.

Next, we computed the correlations between the average music–perceptual ratings of the 34 musical excerpts and the average Perceptual-MCA scores of the colors people associated with the same musical excerpts (Figure 3(a)). Holm's (1979) method was used to control the family-wise error rate, implemented in the “psych” R package (Revelle, 2016). There were statistically significant correlations between the music–perceptual features and the Perceptual-MCAs for 6 of the 15 music–perceptual features. For example, *louder, punchier* musical excerpts were significantly correlated with *more saturated* colors—*loud: r*_*s*_(32) = .642; *punchy: r*_*s*_(32) = .591, *redder* colors—*loud: r*_*s*_(32) = .772; *punchy: r*_*s*_(32) = .643, and *darker* colors—*loud: r*_*s*_(32) = −.557; *punchy: r*_*s*_(32) = −.517. Such correlations show that there are indeed strong perceptual-level music-to-color correspondences. We analyze and discuss the nature of these correlations in more detail in the subsection “Results of dimensional compression of emotion-related scales” later. Figure 3(b) and (c) will be discussed in the subsections “Results of higher level emotional correlations in music-to-color associations” and “Results of correlations between latent Parafac factors and associated colors,” respectively.

#### Discussion

There were notable differences between the present results and the analogous analyses for the more restricted classical musical excerpts previously reported (Palmer et al., 2013, 2016). First, we found strong correlations between musical features and the *red/green* dimension of the associated colors in the present data, which were not previously found for classical orchestral music by Bach, Mozart, and Brahms (Palmer et al., 2013) or for classical piano melodies by Mozart (Palmer et al., 2016). Moreover, there were no significant music–perceptual correlations with the *yellow/blue* dimension, which previously yielded highly significant effects for the classical music. These differences in the nature of hue mappings show that music-to-color associations can be quite distinct with different samples of music.

It is possible that these differences arise because different emotions tend to be expressed in different kinds of music. In particular, Palmer et al.'s (2013, 2016) classical music sample varied more along a *happy/sad* scale, which tends to correlate with *yellow/blue* color variations, whereas the present sample varied more along an *agitated/calm* scale, which tends to correlate with *red/green* color variations. These issues will be assessed empirically in the subsections “Testing the emotional mediation hypothesis” and “Results of dimensional compression of emotion-related scales,” where we consider evidence for emotional mediation.

A second contrast with previous findings concerns differences in the color associations for different tempi. Similar to previous reports for classical music, we found that more saturated colors were selected for faster music. However, unlike previous reports for classical music in which faster tempi and greater note densities—which have similar effects on color associations despite being musically distinct—were associated with lighter, yellower colors (Palmer et al., 2013, 2016), here we found that faster-rated music was associated with darker, redder colors. (Note that some of the latter correlations did not reach significance after correcting for multiple comparisons.) These differences may be due to the fact that faster tempi are correlated with different patterns of other musical features in the different sets of musical samples. For instance, in the present sample, many fast-paced selections were also judged to be *heavy* (*r*_*s*_ = .455) and *punchy* (*r*_*s*_ = .665), whereas the corresponding relations were likely absent in the well-controlled, synthesized, single-line piano melodies by Mozart (Palmer et al., 2016). Different patterns of musical features may also differentially interact to produce different emotion-related experiences (Eerola, Friberg, & Bresin, 2013; Juslin & Lindström, 2010; Lindström, 2003, 2006; Schellenberg, Ania, Krysciak, & Campbell, 2000), hence modulating the types of colors chosen as going with the music. Understanding how musical features interact and map to emotion, and how this may affect color choices, is a complex question that could be addressed using structural equation modeling given a large enough (*n* > 100) sample size of musical excerpts. This is an open area for future research.

### Results of Higher Level Emotional Correlations in Music-to-Color Associations

To examine whether the systematic associations between the music–perceptual and color–appearance dimensions could be mediated by emotion, we conducted corresponding analyses of higher level emotion-related aspects of music-to-color associations. First, we computed the across-subject averages of the 10 music–emotion ratings (from Task A3) for each of the 34 musical excerpts. Next, we correlated each of the average music–emotion ratings with the four Perceptual-MCAs of the colors chosen as going best/worst with the music, analogous to those defined in the previous section. These correlations reflect how the emotional properties of the music correspond to the properties of the colors chosen as going best/worst with them: for example, the extent to which people chose colors that were *more saturated, darker*, and *redder* when listening to *more agitated*-sounding music than when listening to *calmer*-sounding music. The results, plotted in Figure 3(b), show 18 significant correlations. Indeed, every one of the 10 emotion-related scales except for *like/dislike* shows at least one significant correlation at the .05 level using Holm's (1979) method.

#### Discussion

The significant correlations between the emotional content of the music and the perceptual dimensions of the colors picked to go with the music constitute initial evidence that the emotional mediation hypothesis is viable for the expanded sample of 34 diverse musical excerpts. It is also noteworthy that, although none of the 15 music–perceptual features produced a significant correlation with the *yellow/blue* color–appearance dimension (Figure 3(a)), two of the 10 emotion scales did: *happy/sad* (+.55) and *warm/cool* (+.72) (Figure 3(b)). This result shows that, at least in our particular sample of music and colors, emotional mediation accounts for more variability in the *yellowness/blueness* of people's color choices than any single music–perceptual feature we studied.

Further scrutiny of Figure 3(b) reveals different patterns of correlation between music emotions and color appearances. These patterns of correlation can be qualitatively clustered into two sets, with Set 1 split into two subsets. In Set 1A, *spicy, loud, agitated, complex* sounding music was consistently associated with *more saturated, redder, darker* colors. In Set 1B, *appealing, harmonious, liked* music was consistently paired with *lighter* colors that tended to be a bit *greenish* and somewhat *desaturated*. In Set 2, *happy, whimsical, warm* sounding music was consistently associated with *more saturated, yellower* colors. This is in contrast to Sets 1A and 1B, where the emotion-related scales were unrelated to the *yellow/blueness* of color choices.

It is important to note that the correlations for Set1B are nearly opposite to those for Set 1A. If we had plotted the correlations for Set 1B using reversed polarities of the same features (i.e., *disgusting/appealing, dissonant/harmonious*, and *disliked/liked*), the pattern for Set 1B would look qualitatively similar to that for Set 1A. Thus, the patterns of correlations between music emotions and color–appearance dimensions represented in Figure 3(b) appear to reveal two qualitatively different patterns of color choices: one for Set 1 (including both Set 1A and Set 1B) in which more *agitated, spicy, loud, complex, disgusting, dissonant, disliked* music elicits *more saturated, darker, redder* color choices, and one for Set 2, in which more *whimsical, happy, warm* music elicits *more saturated, yellower* color choices.

### Testing the Emotional Mediation Hypothesis

#### Results of correlating music–emotional content and associated colors

**Figure 4.**
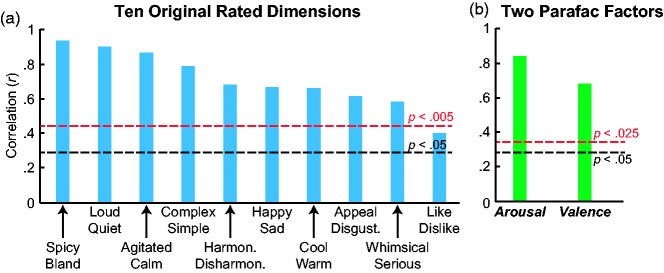
Correlations between the average emotion-related ratings of the 34 musical excerpts and the weighted average emotion-related ratings of the colors chosen as going best/worst with the musical excerpts (EMCAs). (a) Correlations for the 10 originally rated emotion scales. (b) Analogous correlations for the two latent factors in the emotion Parafac solution. The black dotted line corresponds to the uncorrected *p* value cut-off, whereas the red dotted line corresponds to the Bonferroni-corrected *p* values for conducting multiple comparisons.

Next, we analyzed whether people picked colors to go with music based on shared emotional content. We did so by examining correlations between the across-subject average emotion ratings of each of the 34 musical excerpts and the weighted average emotion ratings of the colors chosen as going best/worst with the corresponding excerpts (Emotional-MCAs, or EMCAs; Appendix B). These correlations identify the degree to which people chose colors whose emotional associations matched the emotional associations of the music: for example, choosing *happy*-looking colors as going best/worst with *happy*-sounding music and *agitated*-looking colors as going best/worst with *agitated*-sounding music. Consistent with the emotional mediation hypothesis, 9 of the 10 correlations for the rated musical scales were strongly positive and highly significant after adjusting the alpha level using the Bonferroni correction (.05/10 = .005). As evident in Figure 4(a), these nine correlations ranged from a high of .928 (*p* < .0001, one-tailed) for *spicy/bland*^5^ to a low of .584 (*p* = .00018, one-tailed) for *whimsical/serious* and are thus consistent with emotional mediation of some sort. Although not quite as high as the corresponding correlations in the study using classical orchestral music (.89 < *r* < .99, Palmer et al., 2013), they are roughly comparable to those based on single-line piano melodies (.70 < *r* < .92, Palmer et al., 2016), despite the much wider musical variety in the present sample of music.

The 10th comparison was between preferences for the music and preferences for the colors chosen: that is, correlations between the *like/dislike* ratings for the musical excerpts and the *like/dislike* EMCA scores for the chosen colors for each musical excerpts (the rightmost bar in Figure 4(a)). This preference correlation, although positive, was not significant after correcting for multiple comparisons, *r*_*s*_(32) = .406, *p* = .0086 > .005, one-tailed. The evidence that people chose colors they *like/dislike* as going better with music that they *like/dislike* is thus quite weak, at least for this sample of music, colors, and Western participants.

#### Results of partialling out emotion-related associations

We have now reported evidence for both direct perceptual associations and emotionally mediated associations, but it is unclear that the degree to which the higher level correlations from music emotions to colors (Figure 3(b)) can explain the lower level correlations from music perceptions to colors (Figure 3(a)) versus the degree to which they are independent. A pure version of the direct perceptual link hypothesis (i.e., that all music-to-color associations are due to direct, low-level mappings) implies that the perceptual-feature correlations in Figure 3(a) will be *unsystematically affected* after partialling out the contribution of emotional associations (Emotional-MCAs, calculated earlier). A pure version of the emotional mediation hypothesis (i.e., that all music-to-color associations are due to higher level emotional associations) implies that all significant perceptual-feature correlations in Figure 3(a) will be *eliminated* after partialling out the contributions of all covarying emotional associations.

The partial correlation results support the strong form of the emotional mediation hypothesis. All of the music–perceptual correlations in Figure 3(a) were reduced to nonsignificance after removing the emotional effects in Figure 3(b) and controlling for family-wise error rate using Holm's (1979) method: −.568 ≤ *r*_*s*_ ≤ +.489, *p* > .05 (Figure S2(a)).

### Results of Dimensional Compression of Emotion-Related Scales

#### Latent emotion-related factors of the colors and music

To better understand the shared emotional content of the music–color associations, we used the Parafac model (Harshman, 1970) to discover the latent factors underlying the emotion ratings from Tasks A2 and A3. Parafac was performed jointly on both the color–emotion and the music–emotion ratings (for details, see Appendix C) because previous studies, in which dimensional reductions for music–emotion ratings and color–emotion ratings were conducted separately, found the emotion-related dimensions of the colors and of the music to be very similar (Palmer et al., 2013, 2016).

We examined the weights for Parafac factor solutions containing 2 to 10 factors. We chose the two-factor solution because of (a) its interpretability, (b) its consistency with previous results (Palmer et al., 2013, 2016), (c) its consistency with the clustering of the 10 emotion scales into just two qualitatively different groups (see the subsection “Results of Higher Level Emotional Correlations in Music-to-Color Associations” and Figure 3(b)), (d) the shape of the scree plot, (e) the results of the core consistency diagnostic, and (f) its consistency with the canonical dimensions of human affect: ***arousal*** (or *activation*) and ***valence*** (or *pleasure*) (e.g., Mehrabian & Russell, 1974; Russell, 1980; Russell & Barrett, 1999). The two-factor Parafac model resulted in a clearly interpretable solution and explained 32.7% of the variation in the data tensor $z_{\mathrm{ijk}}$, which includes variance due to individual differences among the 30 participants.

**Figure 5.**
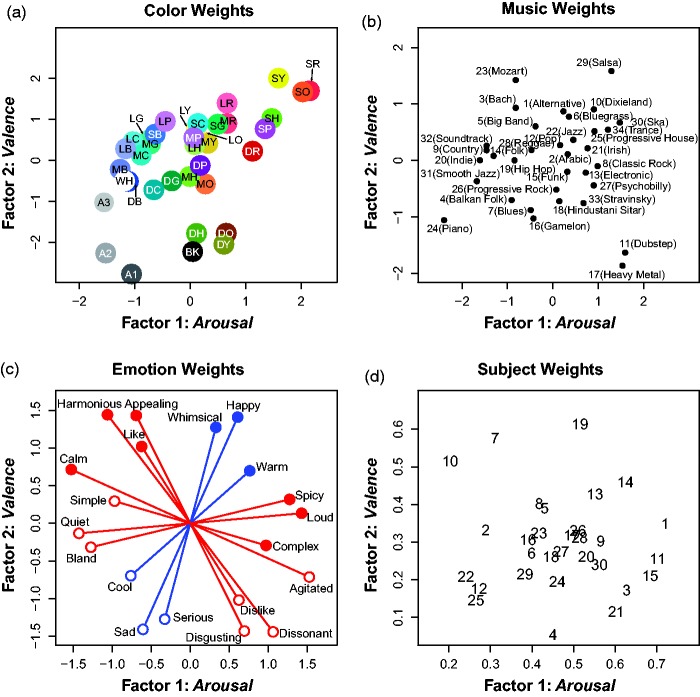
Two-factor emotion Parafac solutions are plotted for the stimulus features (${\text{a}}_{\text{ir}}$) of the 37 colors (a) and 34 musical excerpts (b), the 10 emotion scales (${\text{b}}_{\text{jr}}$) (c), and the individual subject weights (${\text{c}}_{\text{kr}}$) for the 30 participants (d). Because the emotion scales are bipolar, both the actual emotion weights (filled circles) and the implied, inverse of the emotion weights (open circles) are shown. Factor 1 was interpreted as ***arousal*** and Factor 2 as ***valence***. Red lines correspond to Set 1 scales (both Sets 1A and 1B in Figure 3(b)) and blue lines correspond to Set 2 scales (in Figure 3(b)).

The estimated Parafac weights are plotted in Figure 5. The emotion weights in Figure 5(c) show where the emotion-related rating scales are located relative to the axes of the two latent Parafac factors. They are useful for assigning meaning to the factors and labeling them. Factor 1 (along the *x*-axis) is most closely aligned with ratings of *agitated, spicy, loud*, and *complex* on the positive end versus *calm, bland, quiet*, and *simple* on the negative end. We refer to this latent dimension by its affective interpretation, ***arousal***. Likewise, factor 2 (along the *y*-axis) is most closely aligned with ratings of *happy, appealing, whimsical*, and *warm* on the positive end versus *sad, disgusting, serious*, and *cool* on the negative end. We interpreted this latent factor in terms of affect: namely, as ***valence***. For clarity, the term “affect” refers to the two latent factors and the term “emotion” refers specifically to the emotion-related rating scales themselves.

Figure 5(a) plots the weights for the 37 colors and Figure 5(b) for the 34 musical excerpts within the two-dimensional space defined by the latent factors of ***arousal*** and ***valence***. These plots are useful for visualizing the perceptual interrelations among the stimuli with respect to the two factors. For example, saturated red, yellow, and orange are *happy, agitated* colors, high in ***arousal*** and ***valence***, whereas dark grays and blues are *sad, calm* colors, low in ***arousal*** and ***valence*** (Figure 5(a)).

Figure 5(d) plots the subject weights, which are useful for understanding individual differences in the saliences each subject assigned to each factor in choosing the best/worst colors for the music. Most participants weighted ***arousal*** more heavily than ***valence***. We make no attempt to analyze these differences further, however, leaving this topic for future study.

#### Discussion

It is interesting for several reasons that the emotion-ratings data can be well accounted for by two factors, ***arousal*** and ***valence***. First, very similar dimensions were found for music-to-color associations in classical music, even though the emotion ratings were analyzed separately for music and colors (Palmer et al., 2013, 2016). Second, similar affective dimensions have been found in a large segment of the emotion literature, including similarity ratings of facial expressions (e.g., Russell & Bullock, 1985), emotion-denoting words (e.g., Russell, 1980), and ratings of emotionally ambiguous music (e.g., Eerola & Vuoskoski, 2011). Constructionist theories of emotion posit that emotions (e.g., *happiness, sadness*) are constructed from varying degrees of activation along core affective dimensions, typically labeled ***arousal*** (i.e., an energy continuum) and ***valence*** (i.e., a *pleasure/displeasure* continuum) (Kuppens, Tuerlinckx, Russell, & Barrett, 2013; Posner, Russell & Peterson, 2005; Russell, 1980; Russell & Barrett, 1999; Russell, 2003), although other labels have been used (e.g., ***tension*** and ***energy*** instead of ***arousal***; Ilie & Thompson, 2006, 2011). This account contrasts with basic theories of emotion, which suggest that the core experience of emotion can be subdivided into a few, discrete categories (e.g., *happy, sad, angry*, and *fearful*) that are biologically innate and universal (Ekman, 1992; Panksepp, 1998). A great deal of evidence shows that people are usually capable of correctly labeling faces, voices, and instrumental music using basic-emotion categories (e.g., Balkwill, Thompson, & Matsunaga, 2004; Fritz et al., 2009; Scherer, Clark-Polner, & Mortillaro, 2011), but constructionist proponents argue that such experimental manipulations do not rule out the use of variations in arousal and valence to perform such categorizations (Cespedes-Guevara & Eerola, 2018). Indeed, there is considerable evidence in the music–emotion literature for and against both basic and constructionist theories (for in-depth theoretical discussions, see Juslin, 2013 and Cespedes-Guevara & Eerola, 2018; for a review, see Eerola & Vuoskoski, 2013). The present findings suggest that music–emotion and color–emotion ratings can be well described by variations in ***arousal*** and ***valence***, but further work using larger stimulus sets is needed to corroborate our findings.

#### Results of correlations between latent Parafac factors and associated colors

Next, we computed the same correlations as reported in the subsection “Results of Higher Level Emotional Correlations in Music-to-Color Associations” but replaced the across-subject average of the 10 music–emotion ratings for the 34 musical excerpts (Task A3) with the Parafac factor scores corresponding to the 34 music selections: that is, the estimated $a_{\mathrm{ir}}$ scores for ***arousal*** and ***valence***. Figure 3(c) shows that there are several significant correlations between the two latent factors and the color properties of the colors picked to go with the music after correcting for multiple comparisons using Holm's method. Most obviously, more *arousing, agitated* music elicited colors that were more *saturated*—*r*_*s*_(32) = .720, *darker*—*r*_*s*_(32) = −.549, and *redder*—*r*_*s*_(32) = .755, than did *calmer*, less *arousing* music. This pattern corresponds quite closely to that of the emotion-related scales of Sets 1A and 1B in Figure 3(b). In contrast, *positively valenced, happier* music elicited colors that were *lighter, r*_*s*_(32) = .484, and *yellower, r*_*s*_(32) = .466, than did *sadder, negatively valenced* music. This pattern appears to correspond well with the emotion-related scales in Set 2 of Figure 3(b), having positive correlations with both lighter and yellower colors. These results demonstrate that emotion-related associations of music are related to the types of colors people chose to go with the music, even after reducing the 10 original emotion-related scales to just two underlying affective dimensions that reflect the shared variance in the music–emotion and color–emotion ratings.

We also conducted correlational analyses between the music–emotional scales and the color–emotion scales of the colors chosen as going best/worst with the music (i.e., EMCA scores) using the Parafac solutions. These correlations, shown in Figure 4(b), are analogous to those shown in Figure 4(a) but differ in that they replace the across-subject average ratings on the 10 original emotion-related scales for the 34 musical excerpts with those of the two Parafac-based Emotional-MCAs. The formula for calculating these new Parafac-based Emotional-MCAs is identical to that for calculating the Perceptual-MCAs in the subsection “Results of lower-level perceptual correlations in music-to-color associations,” except that the ratings on the four color–appearance dimensions (*saturated/desaturated, light/dark*, etc.) were replaced with the color–emotion Parafac weights representing the emotional associations of the colors that were chosen as going best with the musical selection. These Parafac-based Emotional-MCAs for the 34 musical excerpts were then correlated with the music–emotion Parafac weights for the 34 musical excerpts as represented in Figure 4(b). The results show strong correlations for both ***arousal**, r*_*s*_(32) = .833, *p* < .0001, and ***valence**, r*_*s*_(32) = .678, <.0001. These significant correlations for the present diverse sample of music once again support the conclusions that music-to-color choices are consistent with emotional mediation and that the relevant emotional content is well captured by just the two latent affective factors of ***arousal*** and ***valence***.

#### Results of partialling out the emotional content of the Parafac factors

To better understand how the two latent factors identified by the Parafac analysis relate to the results of the Emotional-MCA partial correlation analysis (Figure 3(b)), we conducted a second partial-correlational analysis examining the correlations between the music–perceptual features and the Perceptual-MCAs after partialling out the factor scores for the 34 music selections: that is, the estimated $a_{\mathrm{ir}}$ scores derived for ***arousal*** and ***valence***. Interestingly, the results showed that, once again, none of the correlations in Figure 3(a) were significant after removing the affective effects in Figure 3(c) and controlling for family-wise error rate using Holm's (1979) method: −.576 < *r* < +.38, *p* > .05 (Figure S2(b)). These findings closely resemble those from the Emotional-MCA partial correlation analysis (Figure S2(a)), implying that the two latent factors capture much of the shared emotional content that is relevant in accounting for the perceptual music–color associations represented in Figure 3(a).

#### Results comparing music–perceptual versus affective predictions of color data

**Figure 6.**
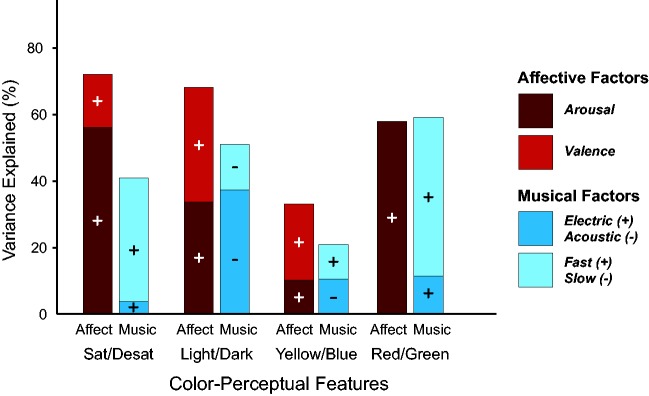
MLR analyses predicting the four color–perceptual values of the colors associated with the musical excerpts (PMCAs) from the Parafac latent factors. The contributions of the first factor entered are represented by the lower segment of each bar, and the polarity of each contribution is indicated by the “+” or “−” sign in that segment.

To compare direct, low-level music–perceptual associations and higher level affective mediation, we further analyzed the ability of each hypothesis to predict the chosen colors along the four color–appearance dimensions using multiple linear regression (MLR) analyses. The music–stimuli weights were entered into the regression and fitted using the ordinary least squares method, with factor 1 always entered into the model first. The results, shown in the red bars of Figure 6, indicate that the two affective factors (***arousal*** and ***valence***) together account for the greatest proportion of variance in the *saturation* (72.2%) and *lightness* (68.3%) dimensions, slightly less in *red/green* (58.3%) and the least amount in *yellow/blue* (33.3%).

For direct comparison, we performed an analogous MLR on the stimuli weights from a corresponding two-dimensional Parafac analysis of the 15 music–perceptual features. The two latent music–perceptual factors were interpreted as ***electronic/acoustic*** and ***fast/slow*** (see S3 Text for further information). The MLR results based on this two-dimensional solution are shown in the blue bars of Figure 6. The affective Parafac solution accounted for more variance than the music–perceptual Parafac solution in *saturation* (40.3%), *lightness* (50.5%), and *yellow/blue* (20.4%) but was about the same in accounting for *red/green* variations (59.1%). The average amount of variance explained by the two affective Parafac factors was thus 58%, about one third more than the average of 42.6% explained by the two music–perceptual Parafac factors.

### The Case for Emotional Mediation

The present research demonstrates that music-to-color associations are better characterized as emotionally mediated (music → emotion → color) rather than direct (music → color) using more conclusive methods than previously employed (Isbilen & Krumhansl, 2016; Lindborg & Friberg, 2015; Palmer et al., 2013, 2016). Although the color–appearance dimensions of the chosen colors were correlated with both music–perceptual features (Figure 3(a)) and emotion-related scales (Figure 3(b)), the lower level correlations were no longer significant after accounting for variance due to the 10 emotion-related scales or after accounting for variance from just the two latent affective dimensions of ***arousal*** and ***valence***. These two latent affective dimensions coincided with the two dimensions of affect that (a) permeate much of the literature on the dimensional structure of human emotions (e.g., Cespedes-Guevara & Eerola, 2018; Mehrabian & Russell, 1974; Osgood, Suci, & Tannenbaum, 1957; Russell, 1980; Russell & Barrett, 1999) and (b) are similar to the dimensions identified previously in research on music-to-color associations using similar methods (Isbilen & Krumhansl, 2016; Palmer et al., 2013, 2016). In addition, the two affective factors were able to predict more variance in the chosen colors than the two music–perceptual factors for all color–appearance dimensions except *red/green* (Figure 6). These results imply that the two latent affective factors are more efficient and effective at predicting the chosen colors than the two-factor, music–perceptual solution.

The interpretation of the present findings converges with previous evidence that emotional/affective mediation also occurs in two additional sets of perceptual mappings: namely, from music to pictures of expressive faces and from pictures of expressive faces to colors (Palmer et al., 2013). In both cases, the emotional mediation correlations were nearly as strong as those found for music-to-color associations with the same emotion-related scales. It is difficult to discern any low-level perceptual features that are common to music, colors, and emotional faces that might reasonably account for the common associative patterns. It is easy to account for these results if they are all mediated by emotion, however.

We hasten to add that the present or previous findings do not rule out the possibility that other kinds of information might also influence music-to-color associations. Semantic effects could arise, for example, if musical excerpts trigger associations with objects or entities that have strong color associations (cf. Spence, 2011). For example, the colors associated most strongly with the Irish selection (Figure 2), which had a prominent bagpipe melodic line, were prominently greens, commensurate with the fact that Ireland is so closely associated with the color green. However, such semantic associations from music to objects/entities to colors may also have emotional components. For example, the blacks and reds chosen predominantly for the heavy metal excerpt (Figure S1) can plausibly be understood as derived from the angry, strong, agitated, and even dangerous sound of the music, given that blacks and dark reds are among the strongest, most angry, agitated, and dangerous looking colors (Palmer et al., 2013; Figure 5).

However, another possibility is consistent with direct associations, namely, through statistical covariation (cf. Spence, 2011). Here, the presumption would be that past experiences cause classical conditioning of direct, cross-modal associations, reflecting the fact that certain kinds of music tend to be heard in visual environments in which certain kinds of colors are predominately experienced. One might frequently hear Salsa music, for example, while seeing (and perhaps eating) tomato-based, Latin American cuisine, such as salsas and enchiladas. However, that explanation seems unlikely to hold for any but a few of the 34 present musical excerpts. Moreover, there is no reason why emotional associations cannot jointly determine music-to-color associations along with these other factors.

### Open Questions

#### Cross-cultural influences

Considering the role of prior experience in music-to-color associations raises the interesting possibility of cultural influences. Would people from different cultures choose the same colors as going with the same music or would they be systematically different? The only existing evidence comes from prior research on music-to-color associations for classical orchestral selections in the United States and Mexico (Palmer et al., 2013). The Mexican data were virtually identical to the U.S. data in every respect. This finding shows that some degree of generalization across cultures is warranted. However, the strength of the generalization is unclear because people in Mexico still have extensive exposure to Western music.

At least two distinct issues underlie the cultural generality of emotional mediation in music-to-color associations. One is the generality of music-to-emotion associations (for a review, see Thompson & Balkwill, 2010). When forced-choice tasks were used with a small number of emotional categories (e.g., choosing among *joy, anger, sadness*, and *peace* while listening to music), the results tend to support generality across cultures (e.g., Balkwill & Thompson, 1999; Balkwill et al., 2004; Fritz et al., 2009). With larger numbers of emotional categories, the results are less clear-cut and depend more heavily on musical familiarity (e.g., Gregory & Varney, 1996). Given that the perception of music is likely influenced by an individual's cultural lens (e.g., Demorest, Morrison, Nguyen, & Bodnar, 2016; McDermott, Schultz, Undurraga, & Godoy, 2016), the true universality of musical emotions is still unclear. Moreover, the theoretical question of whether musical emotions, and human emotions more generally, are represented by discrete, basic categories or from a dimensional, constructionist approach is still debated (e.g., Barrett, Mequita, & Gendron, 2011; Cespedes-Guevara & Eerola, 2018; Juslin, 2013; Ortony & Turner, 1990).

The other issue is the cultural generality of associations between emotions and colors. Most cross-cultural studies take the limited perspective of assessing color on the three dimensions of the semantic differential (Osgood et al., 1957)—namely, evaluation (akin to ***valence***), activity (akin to ***arousal***), and dominance—rather than on basic emotions. Nevertheless, what data exist generally indicate a fair amount of agreement across cultures in their assessment of these dimensions (e.g., Adams & Osgood, 1973; D'Andrade & Egan, 1974). These findings leave open the possibility that music-to-color associations may exhibit some degree of cultural generality.

#### Perceived versus experienced emotion

Another open question is whether the emotion-related correspondence of music and colors that we have identified occurs via perceived emotion (i.e., emotional cognition) or experienced emotion (i.e., emotional feelings or qualia). The distinction is illustrated by the fact that people can clearly perceive the intended emotionality of a piece of music or a combination of colors without actually experiencing that emotion (Gabrielsson, 2002). For instance, it is logically possible to perceive sadness in Hagood Hardy's “If I had Nothing but a Dream” without actually feeling noticeably sadder than before hearing it. We have discussed the 10 rated scales as being “emotion-related” largely to avoid having to commit to whether perceived emotion, felt emotion, or some combination of both is the basis of the emotion-related effects we have reported. It would therefore be desirable to dissociate the relative contributions of perceived versus felt emotion in future cross-modal music-to-color research (e.g., Juslin & Västfjäll, 2008), perhaps through taking relevant physiological measures while participants report on their perception versus experience of emotions (e.g., Krumhansl, 1997) or through studying patient populations, such as alexithymics, who have reduced ability to distinguish between or categorize emotional experiences.

#### Synesthesia

The consistent finding that nonsynesthetes show strong emotion-related effects in cross-modal music-to-color associations, both here and in prior studies (Isbilen & Krumhansl, 2016; Palmer et al., 2013, 2016), warrants further exploration as to whether timbre-to-color synesthetes show similar emotion-related effects. One hypothesis is that the mechanisms producing synesthetic experiences are essentially the same as (or similar to) the mechanisms producing cross-modal nonsynesthetic associations but at an intensity high enough to result in conscious experiences (e.g., Ward et al., 2006). If so, then one would expect that music-to-color synesthetes would also show substantial emotion-related effects in the colors they experience to the same musical excerpts. Indeed, some theorists claim that the primary basis of synesthesia is emotional (e.g., Cytowic, 1989), in which case synesthetes might be expected to show even stronger emotion-related effects than nonsynesthetes. Isbilen and Krumhansl (2016) found that when synesthetes were asked to pick one of eight colors that went best with excerpts of 24 Preludes from Bach's *Well Tempered Clavier*, they chose colors that were consistent with nonsynesthetic color choices. However, because synesthetes were not asked to pick the colors that were *most similar to their synesthetic experiences*—rather, they picked the colors that “best fit the music”—it is unclear whether synesthetic experiences are emotionally mediated, or whether synesthetes are simply capable of making best fitting cross-modal music-to-color choices that are similar to those of nonsythesthetes.

A competing claim is that synesthetic experiences occur via hyperconnectivity from one area of sensory cortex to another (e.g., Ramachandran & Hubbard, 2001; Zamm, Schlaug, Eagleman, & Loui, 2013). By this account, music–color associations in timbre-to-color synesthetes more likely arises from the specific qualities of the sounds (i.e., their timbre, duration, loudness, and pitch) directly activating specific qualities of colors rather than from any higher level, emotion-related attributes. It would be surprising from this perspective if synesthetes showed any emotion-related effects that were not spurious by-products of direct auditory-to-visual mappings.

## Conclusions

The results of this study contribute importantly to the understanding of music-to-color associations in at least four ways.

1. They present robust evidence of emotional mediation of cross-modal music-to-color mappings over a broad range of 34 musical excerpts and 10 emotion-related scales.
2. The present experiment investigated a larger and more diverse set of 15 underlying musical features, many of which have not been previously studied for their color associations (e.g., *loudness, harmony, distortion, beat strength*, and *complexity*, among others).
3. The pattern of results shows that essentially the same emotion-related effects are evident when using a wider range of linguistically labeled scales. Specifically, we found that the 10 emotion-related scales can largely be reduced to the same two latent factors (Figure 5) that are easily identifiable with the affective dimensions of ***arousal*** and ***valence*** discussed in theories of emotion (e.g., Mehrabian & Russell, 1974; Russell, 1980).
4. MLR analyses showed that the two affective factors (***arousal*** and ***valence***) are more effective and efficient in predicting the color–appearance dimensions of music-to-color associations than the corresponding two music–perceptual factors.

Overall, the present results have brought us closer to understanding the role of emotion in people's cross-modal associations to music.

## Supplemental Material

Supplemental Data1 - Supplemental material for Color, Music, and Emotion: Bach to the Blues Click here for additional data file. Supplemental material, Supplemental Data1 for Color, Music, and Emotion: Bach to the Blues by Kelly L. Whiteford, Karen B. Schloss, Nathaniel E. Helwig and Stephen E. Palmer in i-Perception

## Supplemental Material

Supplemental material for Color, Music, and Emotion: Bach to the Blues Click here for additional data file. Supplemental Material for Color, Music, and Emotion: Bach to the Blues by Kelly L. Whiteford, Karen B. Schloss, Nathaniel E. Helwig and Stephen E. Palmer in i-Perception

## Notes

1 We call them “emotion‐related” scales because it is difficult to discriminate categorically between emotional and nonemotional scales. The primary meanings of some dimensions are clearly emotion‐related (e.g., *happy/sad, agitated/calm*), those of others seem to have a significant, but less salient, emotion component (e.g., *strong/weak*, *serious/whimsical*), and those of still others are only marginally related to emotion, if at all (e.g., *simple/complex*, *warm/cool*). All of these dimensions—and, of course, many others—are properly considered “semantic” as well.

2 The “best/worst” paradigm is described in the “Methods” section. For more information, the interested reader is referred to Marley and Louviere (2005).

3 The colors were arranged systematically by hue and cut to allow participants to find appropriate colors easily. The arrangement was the same for every participant on every trial in this study because reversing the up/down and left/right ordering of the colors (by rotating the array through 180°) does not affect results (see Palmer et al., 2013).

4 No attempt was made to validate criteria b, c, or d empirically.

5 We note that the primary meaning of *spicy/bland* indicates a dimension of taste, rather than emotion, but many dictionaries also list emotion‐related synonyms, such as *lively*, *spirited*, and *racy*. We also note that the primary meaning of *warm/cool* is to indicate temperature and that it can also be used to describe colors (where *warm* colors include reds, oranges, and yellows, and *cool* colors include the greens, blues, and purples). Again, most dictionaries also include emotion‐related definitions, such as defining *warm* as “readily showing affection, gratitude, cordiality, or sympathy” and *cool* as “lacking ardor or friendliness” (see http://www.merriam‐webster.com/dictionary). Linguistic terms for music–emotion and color–emotions thus seem to be highly susceptible to metaphorical extensions from other semantic domains.

## Appendix A

From Task C1, we have a matrix that contains the average color–appearance ratings across 48 participants (from Palmer et al., 2013) of the 37 colors in Figure 1 on the color–appearance dimensions *saturated/desaturated, light/dark, red/green*, and *yellow/blue*. Let $x_{\mathrm{cj}}^{C1}$ denote the data from Task C1, that is, $x_{\mathrm{cj}}^{C1}$ is the average rating of the *c*th color on the *j*th color–appearance dimension. From Task A1, we have the three colors from Figure 1 that were judged to be most consistent and most inconsistent with each of the 34 samples of music from Table 1. Let $c_{\mathrm{mk}1}$, $c_{\mathrm{mk}2}$, and $c_{\mathrm{mk}3}$ denote the first, second, and third colors, respectively, that the *k*th subject selected as most consistent (*c*) for the *m*th musical excerpt, and let $i_{\mathrm{mk}1}$, $i_{\mathrm{mk}2}$, and $i_{\mathrm{mk}3}$ denote the corresponding colors that were selected as most inconsistent (*i*). The PMCA score for the *m*th music excerpt on the *j*th color–appearance dimension as judged by the *k*th subject is calculated as

$$PC_{\mathrm{mjk}}=(3x_{(c_{\mathrm{mk}1})j}^{C1}+2x_{(c_{\mathrm{mk}2})j}^{C1}+x_{(c_{\mathrm{mk}3})j}^{C1})/6$$

$$PI_{\mathrm{mjk}}=(3x_{(i_{\mathrm{mk}1})j}^{C1}+2x_{(i_{\mathrm{mk}2})j}^{C1}+x_{(i_{\mathrm{mk}3})j}^{C1})/6$$

$$\mathrm{PMC}A_{\mathrm{mjk}}=PC_{\mathrm{mjk}}-PI_{\mathrm{mjk}}$$

The result of applying the Perceptual-MCA formula is thus to map each music excerpt for each participant into four numbers representing weighted averages of how, *saturated/desaturated, light/dark, red/green*, and *yellow/blue*, the six colors are that were chosen (as the three best and three worst) by each participant for each musical excerpt.

## Appendix B

From Task A1, we have the three colors that were judged to be most and least consistent with each of the 34 samples of music for each subject. Let $c_{\mathrm{mk}1}$, $c_{\mathrm{mk}2}$, and $c_{\mathrm{mk}3}$ denote the first, second, and third colors, respectively, that the *k*th subject selected as most consistent for the *m*th musical excerpt, and let $i_{\mathrm{mk}1}$, $i_{\mathrm{mk}2}$, and $i_{\mathrm{mk}3}$ denote the corresponding colors that were selected as most inconsistent. Let $x_{\mathrm{cjk}}^{A2}$ denote the data from Task A2, that is, $x_{\mathrm{cjk}}^{A2}$ is the rating of the *c*th color on the *j*th emotion scale for the *k*th subject. The Emotional-MCA score ($\mathrm{EMC}A_{\mathrm{mjk}}$) for the *m*th music excerpt on the *j*th emotion scale as judged by the *k*th subject is calculated as

$$EC_{\mathrm{mjk}}=(3x_{(c_{\mathrm{mk}1})j}^{A2}+2x_{(c_{\mathrm{mk}2})j}^{A2}+x_{(c_{\mathrm{mk}3})j}^{A2})/6$$

$$EI_{\mathrm{mjk}}=(3x_{(i_{\mathrm{mk}1})j}^{A2}+2x_{(i_{\mathrm{mk}2})j}^{A2}+x_{(i_{\mathrm{mk}3})j}^{A2})/6$$

$$\mathrm{EMC}A_{\mathrm{mjk}}=EC_{\mathrm{mjk}}-EI_{\mathrm{mjk}}$$

We then take the average Emotional-MCA score for each music excerpt *m* on each emotion scale across the 30 subjects to characterize the higher level emotional content underlying the music-to-color associations. These across-subject average Emotional-MCA scores for each of the 34 musical excerpts were correlated with the across-subject average music–emotion ratings for the corresponding music (see Figure 4(a)).

## Appendix C

The Parafac model is an extension of principal components analysis (PCA; Hotelling, 1933) for tensor data. Unlike the PCA model, the Parafac model is capable of modeling individual differences in multivariate data collected across multiple occasions and/or conditions. Also, unlike the PCA model, which has a rotational freedom, the Parafac model benefits from the intrinsic axis property such that the Parafac solution provides the optimal (and unique) orientation of the latent factors (Harshman & Lundy, 1994). S2 Text in the Supplemental Materials contains further information about the Parafac model and its relation to factor analysis.

Let $x_{\mathrm{cjk}}^{A2}$ denote the data from Task A2, that is, $x_{\mathrm{cjk}}^{A2}$ is the rating of the *c*th color on the *j*th emotion scale as judged by the *k*th subject. Similarly, let $x_{\mathrm{mjk}}^{A3}$ denote the data from Task A3, that is, $x_{\mathrm{mjk}}^{A3}$ is the rating of the *m*th music excerpt on the *j*th emotion scale as judged by the *k*th subject. To preprocess the data for the Parafac model, we removed the mean (across stimuli) rating of each subject on each scale

$$\begin{array}{c} {\overset{∼}{x}}_{\mathrm{cjk}}^{A2}=x_{\mathrm{cjk}}^{A2}-\frac{1}{37}\sum_{c=1}^{37}x_{\mathrm{cjk}}^{A2}\text{ and }{\overset{∼}{x}}_{\mathrm{mjk}}^{A3}=x_{\mathrm{mjk}}^{A3}-\frac{1}{34}\sum_{m=1}^{34}x_{\mathrm{mjk}}^{A3} \end{array}$$

and then standardized each emotion scale to have equal influence

$$z_{\mathrm{cjk}}^{A2}={\overset{∼}{x}}_{\mathrm{cjk}}^{A2}/s_{j}\text{ where }s_{j}^{2}=\frac{1}{\left(37)(30\right)}\sum_{c=1}^{37}\sum_{k=1}^{30}[{\overset{∼}{x}}_{\mathrm{cjk}}^{A2}{]}^{2}$$

$$z_{\mathrm{mjk}}^{A3}={\overset{∼}{x}}_{\mathrm{mjk}}^{A3}/s_{j}\text{ where }s_{j}^{2}=\frac{1}{\left(34)(30\right)}\sum_{m=1}^{34}\sum_{k=1}^{30}[{\overset{∼}{x}}_{\mathrm{mjk}}^{A3}{]}^{2}$$

Next, we combined the standardized ratings to form $z_{\mathrm{ijk}}$, which represents the *k*th subject's rating of the *i*th stimulus (color or music) on the *j*th emotion scale. We fit the model using the multiway R package (Helwig, 2018) in R (R Core Team, 2018) using 1,000 random starts of the alternating least squares algorithm with a convergence tolerance of 1 × 10^−12^.
