# Supplementary material for: Color, Music, and Emotion: Bach to the Blues
Source: Iperception. 2018 Nov 11;9(6):2041669518808535. doi: 10.1177/2041669518808535 (PMC6240980; doi:10.1177/2041669518808535)
Supplement: Supplemental material for Color, Music, and Emotion: Bach to the Blues [file Supplemental_Material.pdf]

Color, Music, and Emotion: Bach to the Blues

Kelly L. Whiteford, Karen B. Schloss, Nathaniel E. Helwig, and Stephen E. Palmer

Supplementary Materials

Supplementary Text

S1 Text: Selection of Emotion-Related Scales

Prior to conducting the main experiment, a pilot study was conducted with 28 different participants to select the emotion-related scales we would use in the color rating task (A2) and the music rating task (A3). We first compiled a set of 40 emotion-related scales (see Table S3 below) by choosing any term we found in the emotion literature that seemed potentially relevant to music and/or color in the experimenters' collective judgment (e.g., Rentfrow et al., 2012; Scherer, 2005; Zentner, Grandjean, & Scherer, 2008) and by adding scales that we did not find in the literature but thought might be relevant. One group of 14 pilot participants sorted 40 cards, each containing the name of an emotion-related scale (e.g., *happy/sad, full/thin, spicy/bland*), into 12 or fewer categories based on their similarity with respect to color. Then they rated the relevance of each of the 40 scales with respect to color on a continuous line-mark scale labeled "Not Relevant" (-200) on the left-end and "Very Relevant" (+200) on the right-end. A separate group of 14 participants completed the same card-sorting and relevance-rating tasks, but with respect to music instead of color. To arrive at the final list of emotion-related scales for the main experiment, we first eliminated any scales whose average relevance rating was less than zero with respect to either music or color. We also eliminated any remaining scale that was too closely related to any other scale, as measured by an occurrence frequency greater than 9 (of 14) in either of the card sorting tasks. Ten emotion-related scales were thus identified

as being relevant to both color and music without being judged as too similar via the card-sorting task (see Table 2(b)).

S2 Text: Parafac of the 10 Emotion-Related Scales

To find the latent dimensions of the ratings in z_{ijk}, we could have applied classic factor analysis (FA) in two different ways: (i) separately to each subject's data *I* stimuli by *J* emotion scales data matrix, or (ii) to the *I* stimuli by *J* emotion scales data matrix obtained by averaging the ratings across all subjects. However, neither of these options is ideal. The first approach is flexible, i.e., provides a unique decomposition of each subject's data, but lacks a coherent way of comparing the latent dimensions of different subjects. The second approach is simpler to interpret but lacks the flexibility to explore individual differences in the latent dimensions. Furthermore, both approaches have a rotational indeterminacy, which implies that the latent dimensions could be rotated without changing the model fit.

The Parafac model extends the classic FA model by leveraging the individual differences in the data to solve the rotational indeterminacy problem inherent to classic FA models. The Parafac model assumes that the standardized ratings have the form

$$z_{ijk} = \sum_{r=1}^{R} a_{ir} b_{jr} c_{kr} + e_{ijk} \quad (10)$$

where a_{ir} gives the weight of the *i*-th stimulus on the *r*-th factor, b_{jr} gives the weight of the *j*-th emotional scale on the *r*-th factor, c_{kr} gives the weight of the *k*-th subject on the *r*-th factor, and e_{ijk} is the error term (i.e., the portion of z_{ijk} that cannot be explained by the Parafac model structure). Note that the Parafac model assumes that the same latent dimensions explain each subject's ratings, but each subject has a unique set of scores on the latent dimensions (captured by the c_{kr} weights). Furthermore, note that if we had a single subject (K = 1), then we can

assume that $c_{kr} = 1$ and the Parafac model would reduce to the classic PCA / FA model.

S3 Text. Parafac Analysis of the 15 Music-Perceptual Features

Parafac was performed to reduce the 15 music perceptual features to a smaller number of interpretable factors. Let x_{mjk}^{A3} denote the data from Task A3, i.e., x_{mjk}^{A3} is the rating of the *m*-th music selection on the *j*-th music-perceptual feature as judged by the *k*-th subject. To preprocess the data for the Parafac model, we removed the mean rating of each subject on each feature across the 34 musical excerpts

$$\tilde{x}_{mjk}^{A3} = x_{mjk}^{A3} - \frac{1}{34}\sum_{m=1}^{34} x_{mjk}^{A3}$$

and then standardized each music-perceptual feature to have equal influence

$$z_{mjk}^{A3} = \tilde{x}_{mjk}^{A3}/s_j \quad \text{where} \quad s_j^2 = \frac{1}{(34)(15)}\sum_{m=1}^{34}\sum_{k=1}^{15}\left[\tilde{x}_{mjk}^{A3}\right]^2$$

Next, we combined the standardized ratings to form z_{ijk}, which represents the *k*-th subject's rating of the *i*-th stimulus on the *j*-th music-perceptual feature.

The Parafac model assumes that the standardized ratings have the form

$$z_{ijk} = \sum_{r=1}^{R} a_{ir}b_{jr}c_{kr} + e_{ijk}$$

where a_{ir} gives the weight of the *i*-th musical stimulus on the *r*-th factor, b_{jr} gives the weight of the *j*-th music-perceptual feature on the *r*-th factor, c_{kr} gives the weight of the *k*-th subject on the *r*-th factor, and e_{ijk} is the error term (i.e., the portion of z_{ijk} that cannot be explained by the Parafac model structure).

We fit the model using the multiway R package (Helwig, 2018) using 1000 random starts of the alternating least squares algorithm with $R = 2$ to 15 factors. The convergence tolerance was set to $1*10^{-12}$ We chose the 2 factor solution because of (a) its interpretability, (b) the shape

of the scree plot, and (c) the results of the core consistency diagnostic. The two-factor Parafac model explained 39.98% of the variation in the data tensor z_{ijk} and resulted in a moderately interpretable solution. All other factor solutions were complex and difficult to interpret in terms of the underlying structure. The music-perceptual weights for the two-factor solution are plotted in Figure S3(b). They reveal that the two latent factors underlying the music-perceptual data can perhaps best be interpreted as ***electronic /acoustic*** (factor 1) and ***fast /slow*** (factor 2).

Figure S3(a) plots the Parafac stimulus weights for 34 musical excerpts (i.e., a_{ir}), which are useful for visualizing the perceptual interrelations among the stimuli; Figure S3(b) plots the music-perceptual weights (i.e., b_{jr}), which are useful for assigning meaning to the factors; and Figure S3(c) plots the subject weights (i.e., c_{kr}), which are useful for understanding individual differences in the saliences each subject assigned to each factor. We note that although we have named these composite, latent factors with the same labels as two of the originally rated features (***electronic /acoustic*** and ***fast /slow***), we use bold italics to indicate that they are not actually the same because the latent factors include weightings from the entire dataset from task B1, including other rated features. The musical selections represented in Figure S3(a) show a slightly negative bias for the music that is rated as *electronic* tends to be rated as *slower* (e.g., House and Dubstep), whereas the music that is rated as *acoustic* tends to be rated as *faster* (e.g., Dixieland, Mozart, and Jazz). This negative correlation is presumably inherent in the musical sample of 34 selections we studied. Third, the more extreme individuals represented in Figure S3(c) showed a negative correlation in their weighting structure, tending to emphasize ***electronic /acoustic*** over ***fast /slow*** or ***fast /slow*** over ***electronic /acoustic***.

Table S1. CIE 1931 values and Munsell values for the 32 chromatic colors and CIE 1931 values for the five achromatic colors (CIE Illuminant C). (Table from Palmer et al., 2013)

Color		x	y	Y	Hue	Value/Chroma
Red	Saturated	0.549	0.313	22.93	5 R	5/15
	Light	0.407	0.326	49.95	5 R	7/8
	Muted	0.441	0.324	22.93	5 R	5/8
	Dark	0.506	0.311	7.60	5 R	3/8
Orange	Saturated	0.513	0.412	49.95	5 YR	7/13
	Light	0.399	0.366	68.56	5 YR	8/6
	Muted	0.423	0.375	34.86	5 YR	6/6
	Dark	0.481	0.388	10.76	5 YR	3.5/6
Yellow	Saturated	0.446	0.472	91.25	5 Y	9/12
	Light	0.391	0.413	91.25	5 Y	9/6.5
	Muted	0.407	0.426	49.95	5 Y	7/6.5
	Dark	0.437	0.450	18.43	5 Y	5/6.5
Chartreuse	Saturated	0.387	0.504	68.56	5 GY	8/11
	Light	0.357	0.420	79.90	5 GY	8.5/6
	Muted	0.360	0.436	42.40	5 GY	6.5/6
	Dark	0.369	0.473	18.43	5 GY	4.5/6
Green	Saturated	0.254	0.449	42.40	3.75 G	6.5/11.5
	Light	0.288	0.381	63.90	3.75 G	7.75/6.25

	Muted	0.281	0.392	34.86	3.75 G	6/6.25
	Dark	0.261	0.419	12.34	3.75 G	3.75/6.25
Cyan	Saturated	0.226	0.335	49.95	5 BG	7/9
	Light	0.267	0.330	68.56	5 BG	8/5
	Muted	0.254	0.328	34.86	5 BG	6/5
	Dark	0.233	0.324	13.92	5 BG	4/5
Blue	Saturated	0.200	0.230	34.86	10 B	6/10
	Light	0.255	0.278	59.25	10 B	7.5/5.5
	Muted	0.241	0.265	28.90	10 B	5.5/5.5
	Dark	0.212	0.236	10.76	10 B	3.5/5.5
Purple	Saturated	0.272	0.156	18.43	5 P	4.5/17
	Light	0.290	0.242	49.95	5 P	7/9
	Muted	0.287	0.222	22.93	5 P	5/9
	Dark	0.280	0.181	7.60	5 P	3/9
Achromatic	Black	0.310	0.316	0.30		
	Dark gray	0.310	0.316	12.34		
	Med Gray	0.310	0.316	31.88		
	Light Gray	0.310	0.316	63.90		
	White	0.310	0.316	116.00		

Table S2. Details of musical excerpts. Start and stop times are embedded in YouTube links, where possible, but otherwise must be manually stopped after 15 s.

Genre-Excerpt	Artist	Album	Title	Start Time	
				Album	YouTube
Alternative	Metric	*Black Sheep - Single*	Black Sheep	3:39	3:40
Arabic	Simon Shaheen	*Turath*	Bashraf Farahfaza	4:36	4:36
Bach	Johann Sebastian Bach	*Bach: Brandenburg Concertos Nos 1-6*	Brandenburg Concerto No. 5, I a	0:00	0:00
Balkan Folk	Beirut	*Gulag Orkestar*	Canals of Our City	0:44	0:44
Big Band	Glenn Miller	*The Essential Glenn Miller*	String of Pearls	0:08	0:08
Bluegrass	Doc Watson	*The Essential Doc Watson*	Beaumont Rag	0:01	0:03
Blues	Albert King	*The Definitive Albert King On Stax*	Blues Power	2:36	2:36
Classic Rock	BBM	*Around the Next Dream*	City of Gold	1:56	1:56
Country Western	George Jones	*Cold Hard Truth*	Choices	0:00	0:00
Dixieland	Firehouse Five Plus Two	*Firehouse Five Plus Two Story*	Everybody Loves My Baby	0:04	0:04
Dubstep	Bar 9	*UKF Dubstep 2010*	Piano Tune	0:27	0:27
Eighties Pop	Madonna	*True Blue*	Open Your Heart	0:02	0:02
Electronic	LCD Sound System	*This Is Happening*	I Can Change	0:00	0:00
Folk	Cat Stevens	*Tea for the Tillerman*	Where Do The Children Play	2:23	2:23
Funk	Parliament	*Mothership Connection*	Night of the Thumpasorus	0:11	0:11
Gamelon	I Lotring	*Listen, 6th Edition*	Bopong	0:04	NA
Heavy Metal	Slipknot	*All Hope Is Gone*	Psychosocial	0:04	0:04
Hindustani Sitar	Ravi Shankar	*Spirit of India*	Raga Bairagi Todi	10:28	10:28
Hip Hop	J. Cole	*Nothing Lasts Forever*	Nothing Lasts Forever	4:20	4:20
Indie	Pinback	*Pinback*	Loro	0:00	0:00

Irish	The Rogues	*Live in Canada, Eh?*	More Reels	2:06	2:06
Jazz	Dizzy Gillespie	*An Electrifying Evening with the Dizzy Gillespie Quintet*	Salt Peanuts	3:47	3:47
Mozart	Mozart (Philharmonia Orchestra; Vladimir Ashkenazy)	*Listen, 6th Edition*	Piano Concerto in A, K. 488, I a	0:32	0:35
Piano	Hagood Hardy	*Alone*	If I had Nothing But a Dream	0:07	NA
Progressive House	Deadmau5	*Ghosts N Stuff – Single*	Ghosts N Stuff	1:14	0:45
Progressive Rock	Pink Floyd	*The Dark Side of the Moon*	Any Colour You Like	0:27	0:27
Psychobilly	Tiger Army	*Music From Regions Beyond*	Pain	2:19	2:19
Reggae	Bob Marley & The Wailers	*Legend (Remastered)*	Satisfy My Soul	0:00	0:05
Salsa	Sonora Carruseles	*Al Son de los Cueros - Hits de la Salsa, Cumbia & Boogaloo*	Al son de los Cueros	2:11	2:11
Ska	Streetlight Manifesto	*Everything Goes Numb*	Here's to Life	0:05	0:05
Smooth Jazz	Kenny G	*Kenny G: Greatest Hits*	Sentimental	1:50	1:50
Soundtrack	Yann Tiersen	*Amélie (Original Soundtrack)*	La Valse D'Amelie (Version orchestre)	0:29	0:30
Stravinsky	Stravinsky (Altanta Symphony Orchestra; Yoel Levi)	*The Rite of Spring Pulcinella Suite*	Rite of Spring; Part 1: Dances of the Adolescent Girls (Track 2)	0:32	NA
Trance	Darude	*Before the Storm*	Feel the Beat	3:14	3:05

Table S3. The 40 emotion-related scales from the pilot study from which the 10 emotion-related scales were selected for the main experiment.

**Emotion-Related Scales from Pilot Study**			
Aggressive/Gentle	Dirty/Clean	Painful/Soothing	Slow/Fast
Agitated/Calm	Disgusting/Appealing	Quiet/Loud	Small/Large
Angry/Peaceful	Dissonant/Harmonious	Random/Orderly	Solid/Fluid
Angular/Curved	Dreary/Lively	Relaxing/Exciting	Stale/Fresh
Artificial/Natural	Dull/Inspirational	Rough/Smooth	Static/Movement
Bland/Spicy	Evil/Good	Sad/Happy	Strong/Weak
Blurry/Clear	Expected/Surprising	Scary/Comforting	Tense/Relaxed
Closed/Open	Hard/Soft	Serious/Whimsical	Thin/Full
Constrained/Free	Heavy/Light	Sharp/Blunt	Ugly/Beautiful
Cool/Warm	Jarring/Hypnotic	Simple/Complex	Unrefined/Refined

Table S4. Across-subject agreement for the musicians who rated the music-perceptual features (a) and each of the subjects who rated the emotion-related scales (b). Values correspond to Cronbach's α.

**(a) Music-Perceptual Features**	**α**	
Electric/Acoustic	0.931	
Distorted/Clear	0.939	
Many/Few instruments	0.923	
Loud/Soft	0.910	
Heavy/Light	0.964	
High/Low pitch	0.915	
Wide/Narrow pitch variation	0.887	
Punchy/Smooth	0.985	
Harmonious/Disharmonious	0.959	
Clear/No melody	0.939	
Repetitive/Not-repetitive	0.959	
Complex/Simple rhythm	0.967	
Fast/Slow tempo	0.922	
Dense/Sparse	0.844	
Strong/Weak beat	0.957	
**(b) Emotion-Related Scales**	**α (Music Ratings)**	**α (Color-Ratings)**
Calm/Agitated	0.978	0.945
Spicy/Bland	0.946	0.963
Warm/Cool	0.816	0.967
Appealing/Disgusting	0.902	0.892
Harmonious/Dissonant	0.954	0.864
Loud/Quiet	0.982	0.956
Happy/Sad	0.947	0.949
Whimsical/Serious	0.872	0.945
Complex/Simple	0.912	0.896
Like/Dislike	0.879	0.845

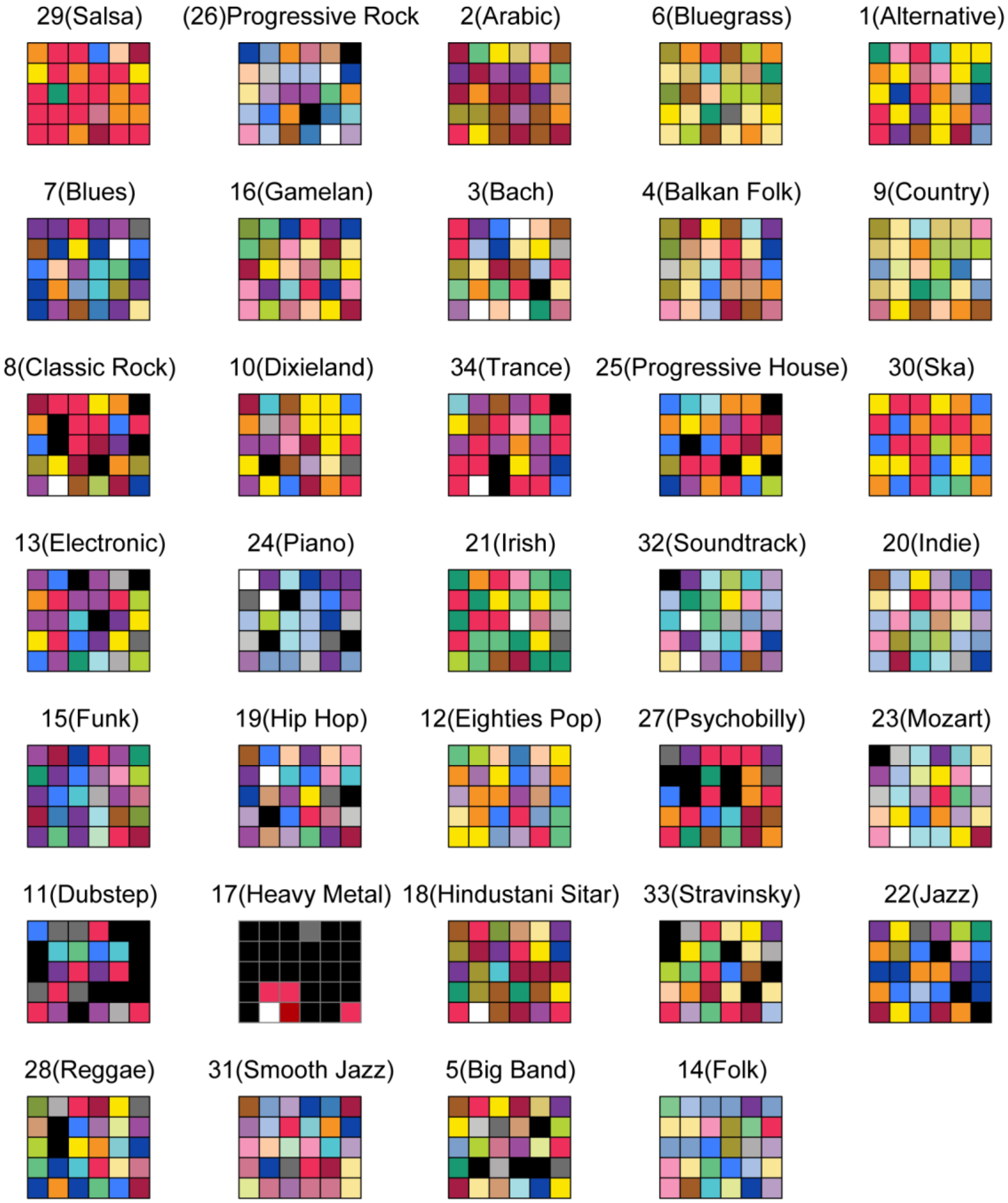

Figure S1. The best-fitting color chosen by each of the 30 participants in the music-to-color association task (A1) for each of the 34 musical selections.

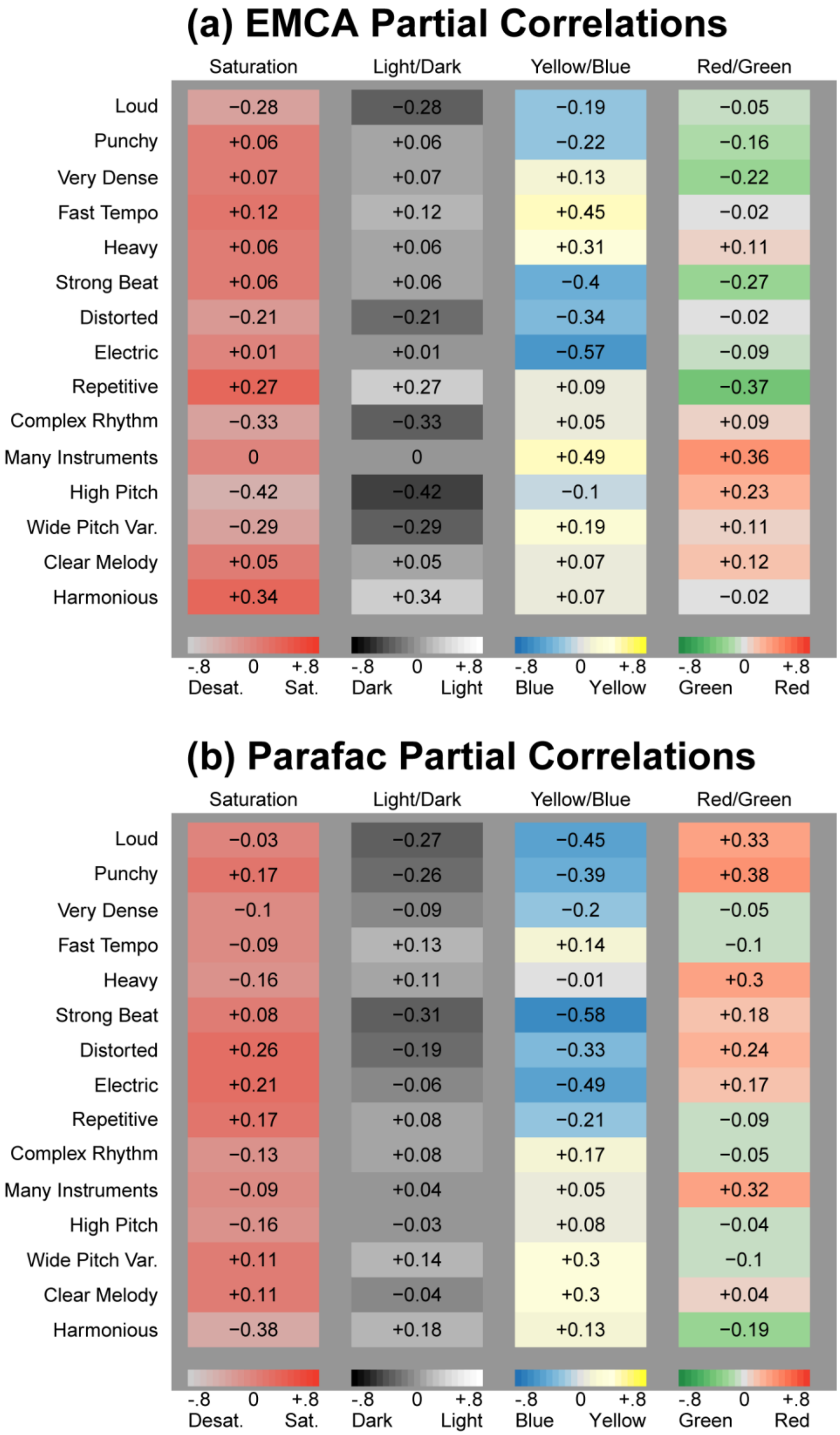

Figure S2. Correlations between the 15 music-perceptual features and the weighted average color-appearance values of the colors picked to go with the music (PMCAs) after accounting for variance due to the EMCAs (a) or after accounting for the variance due to the 2 Parafac latent

affective factors – ***arousal*** and ***valence*** of the music (b). Consistent with the emotional mediation hypothesis, any significant correlations between lower-level perceptual features (in Figure 3(a) of the main text) were no longer significant after accounting for emotion-related content in both cases. Family-wise error rate was controlled using Holm's method.

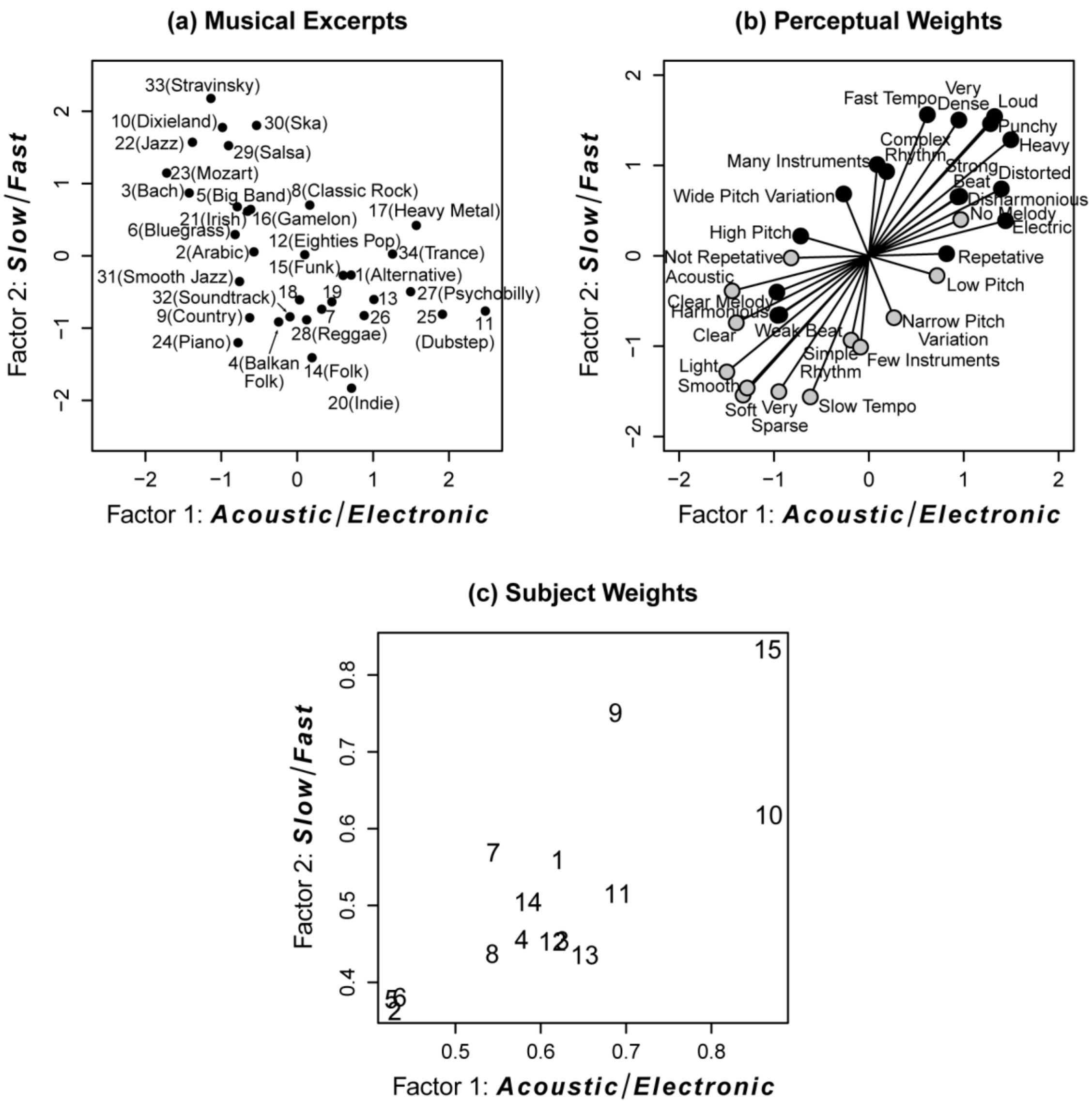

Figure S3. Two-factor music-perceptual Parafac solutions. Panel A plots the weights for the 34 musical excerpts, panel B plots the weights for the 15 music-perceptual features (b_{jr}), and panel C plots the weights for the 30 individual participants (c_{kr}). Because the perceptual features are bi-polar, both the actual perceptual weights (black circles) and the implied, inverse of the perceptual weights (grey circles) are shown. Factor 1 was interpreted as ***electronic /acoustic*** and Factor 2 as ***fast /slow***.

References

Helwig, N. E. (2018). *multiway: Component Models for Multi-Way Data*. R package version 1.0-5. https://CRAN.R-project.org/package=multiway

Palmer, S. E. & Schloss, K.B. (2010). An ecological valence theory of human color preference. *Proceedings of the National Academy of Sciences, 107*, 8877-8882. doi: 10.1073/pnas.0906172107

Rentfrow, P. J., Goldberg, L. R., Stillwell, D. J., Kosinski, M., Gosling, S. D., & Leviti, D. J. (2012). The song remains the same: A replication and extension of the MUSIC model. *Music Perception: An Interdisciplinary Journal, 30*, 161-185. doi:10.1525/mp.2012.30.2.161

Scherer, K. R. (2005). What are emotions? And how can they be measured? *Social Science Information, 44*, 695-729. doi:10.1177/0539018405058216

Zentner, M., Grandjean, D., & Scherer, K. R. (2008). Emotion evoked by the sound of music: Characterization, classification, and measurement. *Emotion, 8*, 494-451. doi:10.1037/1528-3542.8.4.494
